# Supplementary material for: Multi‐dataset identification of innovative feature genes and molecular mechanisms in keratoconus
Source: J Cell Mol Med. 2024 Sep 19;28(18):e70079. doi: 10.1111/jcmm.70079 (PMC11412914; doi:10.1111/jcmm.70079)
Supplement: Supplementary file 2 — Material S2. [file JCMM-28-e70079-s002.zip › 7.GSEA analysis/4.MYRF/KEGG_GLYCOSPHINGOLIPID_BIOSYNTHESIS_GANGLIO_SERIES.html]

Details for gene set KEGG\_GLYCOSPHINGOLIPID\_BIOSYNTHESIS\_GANGLIO\_SERIES[GSEA]

|  || Dataset | MYRF.group.cls#h\_versus\_l |
| Phenotype | group.cls#h\_versus\_l |
| Upregulated in class | h |
| GeneSet | KEGG\_GLYCOSPHINGOLIPID\_BIOSYNTHESIS\_GANGLIO\_SERIES |
| Enrichment Score (ES) | 0.32604408 |
| Normalized Enrichment Score (NES) | 0.93502736 |
| Nominal p-value | 0.54221386 |
| FDR q-value | 1.0 |
| FWER p-Value | 1.0 |
Table: GSEA Results Summary

  

Fig 1: Enrichment plot: KEGG\_GLYCOSPHINGOLIPID\_BIOSYNTHESIS\_GANGLIO\_SERIES      
 Profile of the Running ES Score & Positions of GeneSet Members on the Rank Ordered List

  

| SYMBOL | TITLE | RANK IN GENE LIST | RANK METRIC SCORE | RUNNING ES | CORE ENRICHMENT || 1 | ST3GAL5 | na | 4990 | 0.223 | 0.0159 | Yes |
| 2 | SLC33A1 | na | 6915 | 0.166 | 0.0796 | Yes |
| 3 | ST8SIA1 | na | 8361 | 0.130 | 0.1312 | Yes |
| 4 | ST6GALNAC6 | na | 8485 | 0.127 | 0.2189 | Yes |
| 5 | ST8SIA5 | na | 10125 | 0.094 | 0.2388 | Yes |
| 6 | B3GALT4 | na | 10561 | 0.086 | 0.2876 | Yes |
| 7 | ST6GALNAC5 | na | 11750 | 0.067 | 0.3014 | Yes |
| 8 | ST6GALNAC3 | na | 13115 | 0.049 | 0.2970 | Yes |
| 9 | B4GALNT1 | na | 13266 | 0.047 | 0.3260 | Yes |
| 10 | ST6GALNAC4 | na | 18337 | -0.004 | 0.1827 | No |
| 11 | HEXA | na | 18816 | -0.008 | 0.1749 | No |
| 12 | ST3GAL1 | na | 19414 | -0.015 | 0.1686 | No |
| 13 | GLB1 | na | 22760 | -0.056 | 0.1123 | No |
| 14 | HEXB | na | 22837 | -0.057 | 0.1510 | No |
| 15 | ST3GAL2 | na | 31066 | -0.267 | 0.1055 | No |
Table: GSEA details [plain text format]

  

Fig 2: KEGG\_GLYCOSPHINGOLIPID\_BIOSYNTHESIS\_GANGLIO\_SERIES      
 Blue-Pink O' Gram in the Space of the Analyzed GeneSet

  

Fig 3: KEGG\_GLYCOSPHINGOLIPID\_BIOSYNTHESIS\_GANGLIO\_SERIES: Random ES distribution      
 Gene set null distribution of ES for **KEGG\_GLYCOSPHINGOLIPID\_BIOSYNTHESIS\_GANGLIO\_SERIES**
